# Determination of Propane-1,3-sultone in Workplace Air for Occupational Exposure Assessment

**DOI:** 10.3390/ijerph17041414

**Published:** 2020-02-21

**Authors:** Anna Jeżewska

**Affiliations:** Central Institute for Labour Protection—National Research Institute, Czerniakowska 16, 00-701 Warsaw, Poland; anjez@ciop.pl; Tel.: +48-22-623-4665

**Keywords:** 1,2-oxathiolane 2,2-dioxide, carcinogen, air pollution, workplace air, occupational exsure, chromatography, health sciences, environmental engineering

## Abstract

Propane-1,3-sultone (PS) is an alkylating substance used in the production of polymers, fungicides, insecticides, dyes, and detergents. It is absorbed into the human body by inhalation, digestion, and through the skin; it is also a possible carcinogen. Occupational exposure to this substance may occur on industrial or laboratory contact. In Poland, the maximum allowable concentration (MAC) for PS in workplace air is 7 µg/m^3^. The paper presents a method for determination of PS in workplace air using a gas chromatograph coupled with a mass spectrometer (GC-MS). Air containing PS is passed through a glass tube containing a glass fiber filter and two layers of silica gel. The substance is washed with acetonitrile and the solution obtained analysed using GC-MS. The measuring range for an air sample of 360 L is 0.7 ÷ 14 µg/m^3^. The limit of detection (LOD) is 13 ng/m^3^, limit of quantification (LOQ) is 40 ng/m^3^.

## 1. Introduction

PS (CAS No: 1120-71-4) is a white crystalline solid or colourless liquid with an unpleasant odour. It is soluble in water (100 mg/mL), chloroform, esters, methanol, and aromatic hydrocarbons. The molar mass of PS is 122.14 g/mol and its temperature is boiling 180 °C (39.5 hPa), melting 31 °C. The vapour pressure of PS is 0.48 Pa at 20 °C and 1.3 Pa at 30 °C [1,2,3]. PS is used in the production of dyes (as a colouring aid, starch solubility, and antistatic agent for the treatment of certain polyamide fibres), fungicides and insecticides, resins, vulcanisation accelerators, foaming agents, and others [4,5,6,7,8].

Unfortunately, there is no current data on occupational exposure to this substance. However, PS is known to cause cancer [9,10,11,12,13,14,15]. According to the International Agency for Research on Cancer (IARC), it is a probable human carcinogen (Group 2A) [8]. The European Union classifies PS in carcinogenic category 1B (concentration limit C ≥ 0.01%), as very toxic (harmful if swallowed), and harmful on contact with the skin [3]. Experts from the Scientific Committee on Occupational Exposure Limits [3], the National Institute for Occupational Safety and Health, and Occupational Safety and Health Administration [16] have not set a value for PS hygiene standards, claiming that exposure to this substance should be controlled and limited to the lowest possible level. However, in Poland, the MAC value for PS in workplace air was set at 0.007 mg/m^3^ [2,17]. For each substance for which a MAC value is established, it is necessary to develop a method for its determination in order to enable occupational hygiene laboratories to measure and test its content in workplace air.

PS concentration in air is determined by various methods. Oldeweme and Klockow [18] presented two methods: In the first method, the air containing PS is exposed to scrubbers containing methylisobutylketone as an absorbent. The solution thus obtained is determined using a gas chromatograph (GC) with a flame-photometric detector (FPD) offering selective sulphur detection. In the second method, air containing PS is passed through a denuder, whose internal wall is covered with sodium salt of 2-mercaptobenzothiazole. PS in the form of sodium salt of 2-mercaptobenzothiazolo-S-propanosulfonic acid is extracted and determined using high performance liquid chromatograph (HPLC) with ultraviolet (UV) detection. 

The United States Environmental Protection Agency (EPA) developed a method to analyse volatile organic compounds, including PS, in air. This method consists of putting air samples into a canister; after concentration and thermodesorption, the samples are analysed using a gas chromatograph coupled with a mass spectrometer (GC/MS) [19].

In Poland, PS was determined using a gas chromatograph with a flame ionization detector (GC-FID). The method according to PN-Z-04188:1988 [20] consists of adsorption of PS from air on silica gel, desorption with methanol solution in chloroform, and chromatographic analysis of the obtained solution. The calibration curve range is 0.025 to 0.25 mg/m^3^. This standard did not meet the requirements of the Polish PN standards and was thus removed from its list.

The existing methods do not allow determination of PS in the measurement range currently required for chemical agents (EN 482:2012+A1:2015), i.e., from 1/10 to 2 MAC values (from 0.7 µg/m^3^ to 14 µg/m^3^) [21]. This article presents a new method for determination of PS in workplace air, meeting the specified requirements.

## 2. Materials and Methods 

### 2.1. Equipment

The following equipment was used: gas chromatograph by Agilent Technologies, model 7890A, with mass spectrometer model Agilent Technologies 5975C (Santa Clara, CA, USA) and control program with Wiley 8th Edition mass spectra library, capillary column HP-5MS, 30 m long, internal diameter 0.25 mm with cross-linked poly(5%-diphenyl-95%-dimethylsiloxane) with film thickness of 0.25 μm (Agilent J&W, USA), aspirators AirChek 2000 (SKC Inc., Valley View Road, PA, USA)—range of aspirators 1000 ÷ 3250 mL/min for air sampling, mechanical shaker for extraction of analytes from sorbents: WL-2000, (JWElectronic, Warsaw, Poland), Sartorius BP221S analytical balance (Sartorius AG, Goettingen, Germany) for standard weighing, ARISTON refrigerator-freezer (Merloni Indesit, Lodz, Poland), and EKS series desiccator (WSL, Świętochłowice, Poland) for sample storage. 

### 2.2. Material and Reagents

The following reagents were used in the study: propane-1,3-sulton (Sigma-Aldrich, Co. St. Louis, MO, USA), methanol, toluene, acetonitrile (JT. Baker, Deventer, The Netherlands), steel bottles with helium (Multax, Zielonki-Parcela, Poland). Air sampling materials: ORBO™-43 adsorber tubes containing two layers of XAD-2 resin (100/50 mg) (Supelco, Inc. Bellefonte, PA, USA) and glass tubes containing glass fiber filter and two layers of silica gel (400/200 mg) (SKC, USA cat No. 226-10-03). In addition, approximately 3 mL desorption vessels with nuts and silicone gaskets (equipped with a valve to take the solution without opening it), laboratory glass, and syringes for liquids were used.

## 3. Methodology

### 3.1. Sampling

Air samples containing PS (360 L) were collected in glass tubes containing a glass fiber filter and silica gel (400 mg/200 mg). Sampling time was 6 h. PS was extracted with acetonitrile (1 mL) using the filter and 400 mg of the gel layer, and separately using 200 mg of the protective layer; the solutions obtained were determined chromatographically (GC/MS).

### 3.2. Conditions for Chromatographic Determination

**Figure 1:**
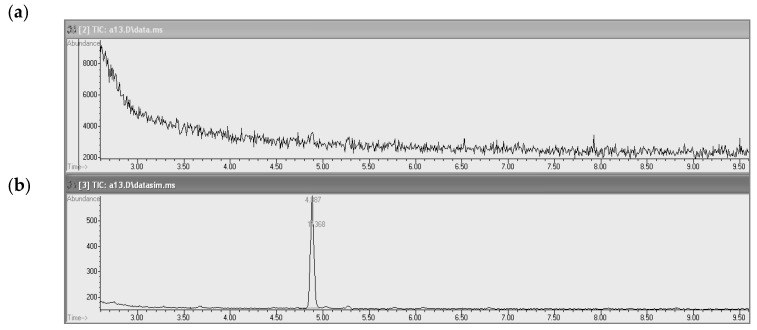
Chromatogram of the PS standard solution in acetonitrile at 0.25 µg/mL (1/10 MAC value) using GC-MS. HP-5MS column (30 m x 0.25 mm; 0.25 µm), programmable temperature, (**a**) SCAN no peak, (**b**) SIM (solvent cut-off 2.5 min): propane-1,3-sultone (t_R_ = 4.887 min).

The analysis was performed using an HP-5MS capillary column (30 m × 0.25 mm; 0.25 μm) at the programmed temperature: initial temperature was 100 °C, temperature increased by 5 °C/min, final temperature 150 °C, with a constant flow of helium through the column at 1 mL/min. The dosing temperature was 250 °C, sample split ratio 10:1, and dosing sample volume 2 µL. Full mass spectra in the range of 35–260 amu were recorded using 70 eV electron ionization. The masses of monitored ions during PS determination in the monitoring mode of selected ions were m/z 30, 58, and 122. The PS chromatogram is shown in Figure 1. Quantitative PS determinations were carried out by monitoring of selected ions.

### 3.3. Examination of Extraction Rate

In order to determine the degree of PS extraction from the filter and silica gel, the following activities were carried out: Two µL of PS solution in acetonitrile at a concentration of 1.11 mg/mL, corresponding to 2.22 µg pure PS, was put in six adsorption tubes containing glass fiber. 360 L of air at a flow rate of 1 L/min was passed through the tubes. PS was then extracted using acetonitrile (1 mL) from the filter and 400 mg of the gel layer, and separately from 200 mg of the protective layer; the solutions obtained were determined chromatographically. The second protective gel layer did not contain the tested substance. PS was also determined in comparative solutions made the same way, but without sorbents. The average extraction factor for PS is 0.99. 

### 3.4. Calibration and Precision

Calibration was determined for six PS standard solutions in acetonitrile at a concentration range of 0.25 to 5.04 µg/mL, which were subjected to chromatographic analysis. Two µL of standard solutions with increasing concentrations were introduced into the chromatograph. The precision of calibration was evaluated on the basis of the analysis of three series of eight working solutions with concentrations of 0.252 (I series), 2.52 (II series), and 5.04 µg/mL (III series). After the chromatographic analysis, standard deviation and coefficient of variation was calculated for each series. 

### 3.5. Validation of the Method

Validation parameters were determined in accordance with the requirements contained in the European Standard EN 482:2012+A1:2015 [21]. The overall approach, including both sampling and analytical methods, was tested by determining the degree of extraction, linearity, sensitivity, precision, and expanded uncertainty. The detection limit (LOD) and limit of quantification (LOQ) were determined from the results of analyses (ten separate peak area measurements with PS retention time) obtained from three separately prepared blank samples. To calculate the detection limit (LOD) value, this relation was used:

(1) $$\mathrm{LOD}\;=\frac{\;3.3\;\cdot {\;s}_{o}}{b},$$

where:

*b*—slope coefficient of the calibration curve, s_o_—standard deviation.
The LOQ value calculated as a multiple of the LOD value, from the following formula:
  (2) LOQ = 3·LOD.

The overall precision of the examination (*V_c_*), which includes laboratory precision for the measurement range and sampling method, was determined from the following formula:

(3) $$V_{c}=\sqrt{V_{z}^{2}+V_{p}^{2}},$$

where:

*V_p_*—is the precision of the sampling device (*V_p_* = ± 5%);
*V_z_*—mean precision of three levels of ranges, which has been calculated using the formula:
  (4) $$V_{z}=\sqrt{\frac{\sum (n_{j}-1)\cdot V_{i}^{2}}{\sum (n_{j}-1)}},$$

where:

*n_j_* is the number of duplicated samples (*n_j_* = 8); and *V_i_* is the variation coefficient for the concentration level.

Overall precision of the examination value (*V_c_*) is taken into account in the calculation of relative total uncertainty. Relative expanded uncertainty was obtained by multiplying the relative total uncertainty with a coverage factor of 2.

## 4. Results and Discussion

The aim of the tests was to determine the conditions for sampling of air containing PS in order to ensure quantitative determination of its content in workplace air. Various filters were tested during the air sampling process: glass fiber, polypropylene, and poly(vinyl chloride) filters. Unfortunately, PS was not retained by these filters, so further testing was conducted using adsorption tubes containing XAD-2 resin and tubes filled with silica gel. During this phase of the study, the ability of the sorbents to retain PS on the measuring bed and the ability of the solvents to extract PS from these sorbents were examined. In the next stage of the study, the amount of air that can be passed through the sampler in order to completely adsorb PS on the measuring bed during individual dosimetry measurements (PN-Z-04008-7:2002) was identified [22]. The research also aimed at checking the conditions of storage of the collected air samples until analysis. 

### 4.1. Testing of Air Sampling Conditions

Due to the very low MAC value for PS, it was necessary to let as much air through the sampler as possible in order to increase the concentration of the substance to be determined on the sorbent. The tests were carried out using ORBO™-43 adsorbing tubes filled with two layers of XAD-2 resin (100/50 mg). Air can be passed through these tubes at a volume flow rate of 2 L/min. Toluene, which was very effective in eluting PS from XAD-2 resin, was used as a desorbent (mean desorption coefficient was 1.05). The results of PS adsorption on XAD-2 resin are presented in Table 1. 

**Figure 2:**
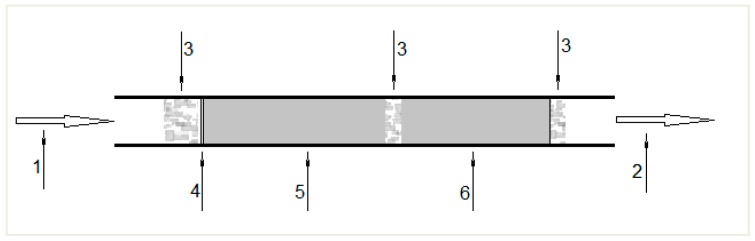
Glass tube for sampling (1) airflow direction, (2) air outlet, (3) glass fiber, (4) glass fiber filter, (5) silica gel first layer (400 mg), (6) silica gel second layer (200 mg) (drawing of the author).

The obtained results indicate that PS penetrates the second layer of XAD-2 resin even if the air volume is reduced from 720 L to 360 L. For this reason, attempts were made to obtain air samples for adsorbent tubes containing glass fiber filter and two layers of silica gel (400/200 mg) (Figure 2). 

Air can pass through these tubes at a volume flow rate of 1 L/min without the pump being halted. Acetonitrile was used as a desorbent, for which the highest desorption coefficient value of 0.99 was obtained in the preliminary tests. The tests for air sampling conditions were carried out on glass fiber contained in adsorbent tubes before the filter; 400 mg a layer of silica gel was introduced successively: pure substance, followed by 12 µL and 28 µL of PS solution in acetonitrile at a concentration of 1.12 mg/mL. 360 L of air was passed through the system at a volume flow rate of 1 L/min. After sampling, 1 mL of acetonitrile was used to extract the substance from the glass fiber and separately from the filter, and from the first (400 mg) and second (200 mg) layers of gel. The results of PS adsorption on glass fiber, glass fiber filter, and two layers of silica gel are presented in Table 2. 

The results obtained show the presence of PS on the filter and 400 mg of silica gel layer.

### 4.2. Determination of Extraction Rate

The study of the degree of PS extraction from the filter and silica gel was carried out as follows: 5 µL of PS solution in acetonitrile at a concentration of 50.4 µg/mL, corresponding to 0.252 µg pure PS, was put in six adsorption tubes with glass fiber. In six more tubes, 2 µL of PS solution was placed in acetonitrile at a concentration of 1.11 mg/mL, corresponding to 2.22 µg PS. In six more tubes, 5 µL of the same solution was added, corresponding to 5.55 µg PS. 360 L of air at a flow rate of 1 L/min were passed through the tubes. PS was then extracted with acetonitrile (1 mL) from the filter and 400 mg of the gel layer, and separately from 200 mg of the protective layer (30 min shaking with a mechanical shaker); the solutions obtained were determined chromatographically. The second protective gel layer did not contain the tested substance. PS was also determined in comparative solutions created the same way, but without sorbents. The results of PS extraction degree tests from silica gel and filter are presented in Table 3. 

The results obtained indicate that acetonitrile can be used for PS extraction from the filter and silica gel. The average extraction factor for the three concentration levels is 0.99.

### 4.3. Calibration and Precision

**Figure 3:**
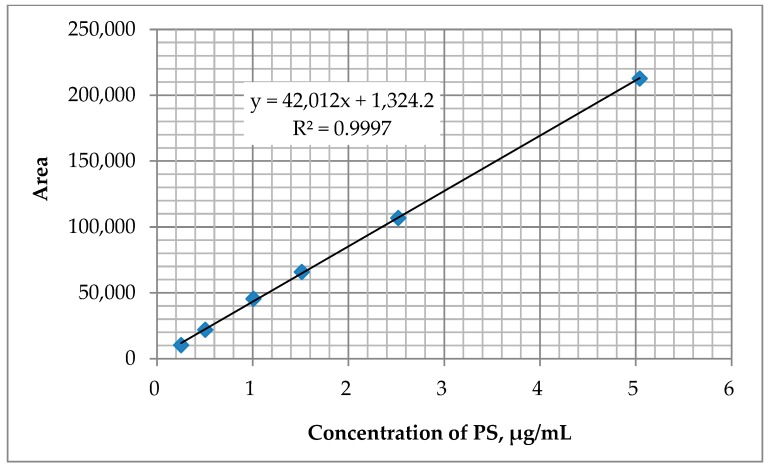
A graph of PS peak area dependence on its concentration in the standard solutions. GC-MS, HP-5MS column, programmable column temperature.

Calibration was determined for six PS standard solutions in acetonitrile at a concentration range of 0.25 to 5.04 µg/mL, which were subjected to chromatographic analysis. Two µL of standard solutions with increasing concentrations were introduced into the chromatograph. Next, a graph of the dependence of the average area of PS peaks on its concentrations in the standard solutions was prepared. The results are shown in Figure 3. 

The slope factor “*b*” of the calibration curve with equation *y = bx + a* characterizing the sensitivity of the method is 42,012. The correlation coefficient *r* characterizing the linearity of the calibration curve is 0.9999.

The precision of calibration was evaluated on the basis of the analysis of three series of eight working solutions at concentrations of 0.252 (I series), 2.52 (II series), and 5.04 µg/mL (III series). The coefficients of variation for subsequent concentration levels were 5.11%; 2.29%, and 1.18%, respectively. The overall precision of the examination determined (according to formula 3) was 5.99%. 

### 4.4. Sample Storage

The stability of the air samples was tested successively after two, five, and seven days of storage in a desiccator and refrigerator. The results showed that the samples stored in the desiccator at room temperature (22 °C ± 2 °C) and in the refrigerator (4 °C ± 2 °C) were stable for at least seven days. The results are shown in Table 4.

### 4.5. Validation

The validation data obtained on the basis of the conducted research are presented in Table 5 and are in accordance with the requirements of EN 482:2012+A1:2015, which are specific to procedures specified for the determination of harmful substances in workplace air. The limit of quantification was 40 ng/m^3^ (for an air sample of 360 L). The relative expanded uncertainty was found to be 26%, which is acceptable for such methods.

## 5. Conclusions

In this study, a suitable sorbent was selected to sample PS in air. A glass tube containing a glass fiber filter and silica gel analysed air samples taken from workplace air, in accordance with the principles of personal dosimetry; this provided the most reliable results for occupational exposure assessment. The conditions for PS determination with GC-MS were selected. A linear calibration curve was obtained within the range studied (0.7 ÷ 14 µg/m^3^). In this respect, the method was validated as per European Standard EN 482:2012+A1:2015 guidelines, meeting the requirements of this standard. This method can be used by occupational hygiene laboratories to determine PS concentrations in workplace air.

## Figures and Tables

**Table 1:** Results of PS adsorption on XAD-2 resin (ORBO™-43)—toluene desorption. GC-MS, HP-5MS column, programmable column temperature.

Air Flow Rate [L/min]	Sampling Time [min]	Approximate Substance Concentration in the Air [mg/m^3^]	Area of PS Peaks in Solutions after Desorption			Quantity of the Substance in Layer II of XAD-2 (in % of the Quantity Determined in Layer I)
			Glass Fiber	Layer I	Layer II	
2	360	pure substance	no peaks	4,346,325	490,610	11
1	360	pure substance	no peaks	11,124,567	1,333,226	12
2	360	0.08	no peaks	1,292,867	211,425	16.4
1	360	0.16	no peaks	1,035,235	36,662	3.5
1	360	0.08	no peaks	538,958	14,463	2.7
1	360	0.04	no peaks	397,602	13,531	3.4

**Table 2:** Results of PS adsorption on glass fiber, glass fiber filter, and silica gel (SKC glass tubes). GC-MS, HP-5MS column, programmable column temperature.

Air Flow Rate [L/min]	Sampling Time [min]	Approximate Substance Concentration in the Air [mg/m^3^]	Area of PS Peaks in Solutions after Extraction with Acetonitrile (1 mL)			
			Glass Fiber	Glass Fiber Filter	Gel Layer I	Gel Layer II
1	60	pure substance	no peaks	47,141	210,340	no peaks
1	360	0.08	no peaks	3629	530,567	no peaks
1	360	0.04	no peaks	3356	231,738	no peaks

**Table 3:** Tests of PS extraction degree from silica gel and filter. GC-MS, HP-5MS column, programmable column temperature.

Mass of PS Applied to the Adsorption Tube [µg]	Average Peak Area of Extracted Solutions	Average Peak Area of the Comparative Solutions	Average Extraction Coefficient
0.252	11,860.3	12,022	0.99
2.22	107,519.5	108,996	0.99
5.55	247,082.6	249,414	0.99

**Table 4:** Results of the stability test of samples containing about 5 µg PS. GC-MS, HP-5MS column, programmable column temperature.

Tube No.	Storage Place	Storage Time [Number of Days]	Average Peak Area of the PS	Two-Tube Average	Standard Deviation
1	desiccator	1	247,687.0	249,692.00	2835.5
2			251,697.0		
1	desiccator	2	263,444.0	253,379.50	14,233.4
2			243,315.0		
1	refrigerator	2	237,593.0	242,193.00	6506.1
2			246,794.0		
1	desiccator	5	246,684.0	249,647.00	4190.3
2			252,610.0		
1	refrigerator	5	246,472.5	247,481.75	1427.3
2			248,491.0		
1	desiccator	7	253,114.5	254,277.00	1644.0
2			255,439.5		
1	refrigerator	7	257,130.0	256,742.50	548.0
2			256,355.0		

**Table 5:** Validation parameters of the method for PS determination.

Parameter	Value
Measurement range	0.7 ÷ 14 µg/m^3^
Sampled air volume	360 L
Range of calibration curve	0.252 ÷ 5.04 µg/mL
Limit of detection (LOD)	4.82 ng/mL (13 ng/m^3^)
Limit of quantitation (LOQ)	14.46 ng/mL (40 ng/m^3^)
Overall precision of examination	5.99%
Relative total uncertainty	13%
Relative expanded uncertainty	26%
